# Continuous vs. Interrupted Multi-Talker Babble Background Noise: Impact on Speech Perception in Bimodal Cochlear Implants

**DOI:** 10.3390/jcm15114232

**Published:** 2026-05-30

**Authors:** Courtney Kolberg, Sarah O. Holbert, Madison K. Graham, Melissa D. DeJong, Cynthia A. Hogan, Aniket A. Saoji

**Affiliations:** 1 Division of Audiology, Department of Otolaryngology-Head and Neck Surgery, Mayo Clinic, Scottsdale, AZ 85259, USAholbert.sarah@mayo.edu (S.O.H.); 2 Division of Audiology, Department of Otolaryngology-Head and Neck Surgery, Mayo Clinic, Rochester, MN 55905, USA; graham.madison@mayo.edu (M.K.G.); dejong.melissa@mayo.edu (M.D.D.); hogan.cindy@mayo.edu (C.A.H.)

**Keywords:** cochlear implants, multi-talker babble, interrupted noise

## Abstract

**Background/Objectives**: Speech perception is measured in quiet and in the presence of background noise, such as multi-talker babble (MTB), to establish cochlear implant (CI) candidacy and measure post-operative outcomes. Testing protocols often use interrupted noise that begins just before and ends shortly after the target sentence, providing insufficient time for the noise-reduction algorithms to activate in the hearing device and provide an improved signal-to-noise ratio (SNR). Furthermore, interrupted noise does not allow the hearing-impaired user to acclimatize to the abrupt onset of noise or to focus on the target sentence, a challenge related to auditory stream segregation. The present study compares speech perception in bimodal CI users across interrupted and continuous MTB noise conditions to highlight performance differences between these two distinct listening environments. **Methods**: Speech perception was evaluated for two different bimodal CI groups. Group 1: This group evaluated an automatic hearing aid program across three environments: quiet, continuous MTB, and interrupted MTB. Group 2: This group evaluated two manual hearing aid programs across the same three environments (quiet, continuous, and interrupted MTB). **Results**: For Group 1, scores in quiet reached 88%, significantly declined in interrupted MTB (38%), but improved in continuous MTB (67%). For Group 2, no significant mean differences were found between interrupted and continuous noise across the two manual programs, though individual variations persisted. **Conclusions**: Based on the findings of this study, continuous noise rather than interrupted noise should be utilized for establishing CI candidacy and evaluating post-implantation speech perception.

## 1. Introduction

**Figure 1:**
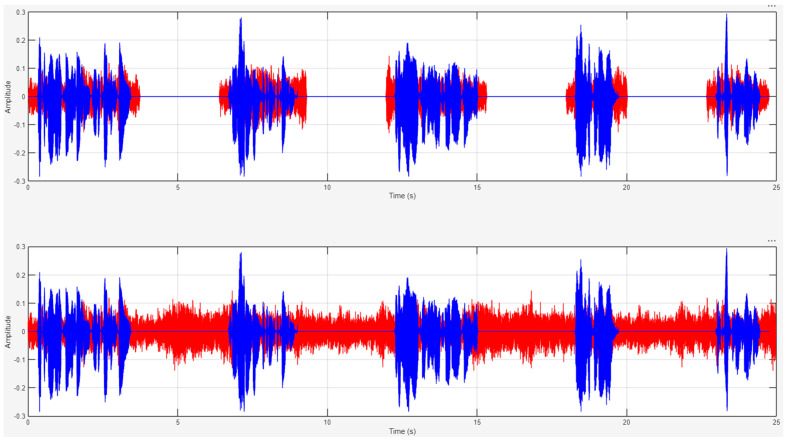
The top panel illustrates the presentation of AzBio sentences (blue) presented in interrupted multi-talker babble (red), while the bottom panel depicts the sentences presented with continuous babble (red).

Speech perception scores measured in the presence of background noise are routinely used to qualify patients for cochlear implant (CI) candidacy and to measure post-operative outcomes. These measures apply to unilateral, bilateral, and bimodal patients (i.e., those using an implant in one ear and a hearing aid in the other). In the United States, speech perception is commonly measured using AzBio sentences [1]. To simulate real-world environments, these tests utilize multi-talker babble (MTB) noise to assess the performance of both hearing aids and cochlear implants. While measuring speech perception in the presence of MTB background noise, the noise onset occurs several hundred milliseconds prior to the presentation of the sentence token. The noise remains active throughout the token duration and is terminated a few hundred milliseconds after the sentence ends. Following each presentation, hearing-impaired listeners repeat the sentence; this cycle is repeated for the duration of the testing session (Figure 1, top panel). For the purposes of this manuscript, this method of testing will be referred to as interrupted noise.

While measuring speech perception scores in the presence of interrupted noise offers an initial glimpse into the challenges faced by hearing-impaired listeners, this method possesses several significant shortcomings. Modern hearing aids and CI speech processors are equipped with sophisticated Automatic Scene Analyzers [2]. These systems are designed to detect when a user transitions from a quiet environment to a noisy one. Upon detecting background noise, the noise reduction algorithms in hearing aids or CI processors undergo a brief adaptation period. They require a few seconds of continuous signal to complete a statistical analysis before the noise suppression is fully engaged [3,4]. Only after this “settling time” can the device effectively deploy advanced noise-reduction strategies, such as adaptive directional microphones [5,6] and/or deep neural network-based noise reduction [7,8,9] that lead to significant improvements in speech perception in the presence of background noise. Clinical speech perception tests often use individual sentences which typically last only 1.0 to 2.0 s. If the background noise only activates during and around these short bursts, the duration of the noise is insufficient for the algorithm to accurately assess the environment, engage the appropriate noise reduction algorithms, and improve the signal-to-noise ratio (SNR).

Furthermore, the auditory system requires a brief period to perform auditory scene analysis [10] and activate top-down gating mechanisms when a hearing-impaired listener enters a real-world noisy environment. This cognitive process allows the brain to suppress competing background noise and prioritize the speaker of interest, which is essential for the “cocktail party effect,” [11,12,13]. However, testing with interrupted noise that starts and stops abruptly alongside speech may not provide enough lead time for the listener to isolate the signal or build a stable mental model of the background noise. Consequently, this sudden acoustic onset does not accurately reflect the natural auditory adaptation that would take place in real-world noisy listening conditions and can be particularly distracting for older hearing-impaired individuals who may have cognitive delays or impaired auditory processing [14].

Taken together, the use of interrupted noise may lead to speech perception in noise scores that underestimate a patient’s true speech understanding. Interrupted noise does not allow time for a hearing device’s advanced noise processing to effectively kick in and optimize the SNR, nor does it enable one to leverage auditory stream segregation, thus overstating the amount of difficulty encountered by hearing-impaired individuals in the noisy listening conditions. The potential for artificially low speech perception in noise scores despite advanced technology may bias clinicians away from recommending such devices.

The purpose of the present study was to evaluate the impact of interrupted versus continuous MTB noise (Figure 1, bottom panel) used for speech perception testing in bimodal cochlear implant users. Unlike the interrupted MTB noise, the continuous MTB background noise is presented without interruption throughout the entire testing block, persisting during the sentence tokens and remaining active while participants repeat their responses until the test terminates. Two groups of bimodal CI users were assessed to determine how these two noise presentation modes affect speech perception, specifically focusing on two factors: (1) the time constants required for the hearing aid’s noise reduction algorithms to activate when in an automatic program, and (2) the role of auditory processing and auditory stream segregation in allowing the listener to cognitively adapt to a stable acoustic environment.

## 2. Methods

**Figure 2:**
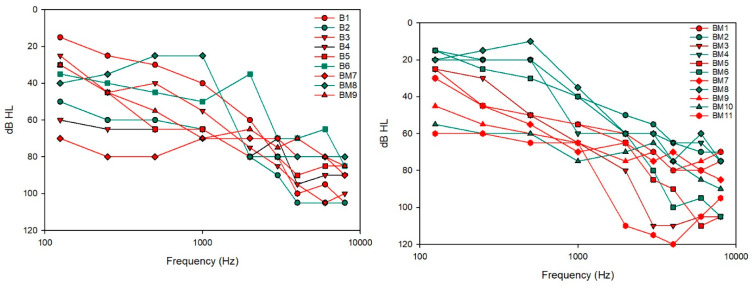
The (**left**) panel displays the audiometric air-conduction thresholds for participants in Group 1, while the (**right**) panel illustrates the corresponding thresholds for participants in Group 2, both measured in the hearing aid ear.

Data for this study was analyzed from two groups of bimodal CI users. Group 1 (N = 9) had a mean age of 81.4 ± 3.9 years. Seven participants had Cochlear Nucleus (Cochlear Limited, Sydney, Australia) devices (1 CI632, 1 CI1022, 4 CI-622, 1 CI522) and two used Advanced Bionics (Advanced Bionics LLC, Valencia, CA, USA) devices (SlimJ/HiFocus). Group 2 (N = 11) had a mean age of 78.3 ± 4.0 years. Seven participants had Cochlear Nucleus devices (3 CI632, 1 CI612, 2 CI-622, 1 CI24RE) and four used Advanced Bionics (3 SlimJ, 1 HiFocus Mid-Scala) devices. Contralateral air-conduction thresholds for both groups are illustrated in Figure 2 (left and right panels, respectively).

All participants were fit with a Phonak Audéo Sphere Infinio 90 receiver-in-canal hearing aid (Stäfa, Switzerland) equipped with power domes during standard clinical visits. On-ear verification was performed to NAL-NL2 targets. Immediately following the hearing aid fitting, both subject groups underwent speech perception testing in 3 conditions: quiet, interrupted MTB noise, and continuous MTB noise. All testing was completed in the bimodal listening configuration. Speech perception was evaluated using one list of twenty AzBio sentences per test condition. Patients were evaluated in a double-walled sound booth. Speech and the MTB noise stimuli were delivered via a single loudspeaker located at 0 degrees azimuth and 1 m from the user. Participants were first presented with several practice sentences to familiarize them with the task and the testing environment.

For Group 1, the hearing aid was set to AutoSense OS, which utilizes machine learning and scene classification to deploy various signal processing and noise reduction strategies, including deep neural network (DNN)-based noise reduction. During testing, the participants’ cochlear implant sound processor was set on a program that utilized their device’s default environmental classifier—either the AutoSense OS for Advanced Bionics users or the SCAN program for Cochlear Limited recipients. Thus, Group 1 utilized automatic programs bimodally. Group 1 was tested across three conditions: AzBio Quiet, AzBio MTB Interrupted, and AzBio MTB Continuous. AzBio sentences were presented at 75 dB SPL in quiet and at a +5 dB signal-to-noise ratio (SNR) during noisy conditions. This specific presentation level was selected to ensure the background noise exceeded the threshold required to activate the deep neural network (DNN)-based noise reduction available in the hearing aid.

Group 2 participants were provided with two manual hearing aid programs: “Calm Situation,” which lacks noise reduction features, and “Spheric Speech in Loud Noise,” which utilizes DNN-based noise reduction and beamforming algorithms. In the CI ear, participants utilized their sound processor’s default environmental classifier—either AutoSense OS (Advanced Bionics) or SCAN (Cochlear). Group 2 underwent speech perception testing across five conditions: AzBio Quiet—Calm Situation; AzBio MTB Interrupted—Calm Situation; AzBio MTB Continuous—Calm Situation; AzBio MTB Interrupted—DNN; and AzBio MTB Continuous—DNN. AzBio sentences were presented at 60 dB SPL in quiet, with noisy conditions set at either 0 dB or +5 dB SNR. These signal-to-noise ratios were tailored to each participant to prevent ceiling or floor effects in the data.

## 3. Results

**Figure 3:**
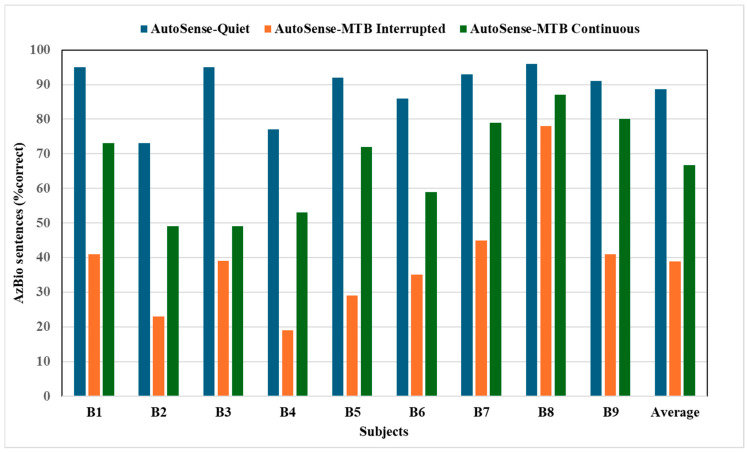
Individual and mean speech perception scores for Group 1 (N = 9) using the AutoSense OS program. Performance is shown in quiet and in the presence of continuous and interrupted multi-talker babble.

Figure 3 illustrates speech perception scores for Group 1 using the AutoSense OS program on the Phonak Audéo Sphere Infinio hearing aid. The mean AzBio sentence score in quiet reached 88%, decreased significantly with the introduction of interrupted MTB noise to 38%, but again improved in the presence of continuous MTB noise, averaging 67%. Therefore, a 29 percentage-point improvement in performance was observed when testing in continuous noise compared to interrupted noise. The data met the assumptions for normality (Shapiro–Wilk test). A one-way ANOVA confirmed a statistically significant difference in sentence recognition across conditions [F (2, 24) = 29.38, *p* < 0.001]. Subsequent pairwise comparisons using the Student–Newman–Keuls post hoc test revealed significant differences between all test conditions (*p* < 0.01), confirming that speech perception was superior in continuous noise relative to interrupted noise.

**Figure 4:**
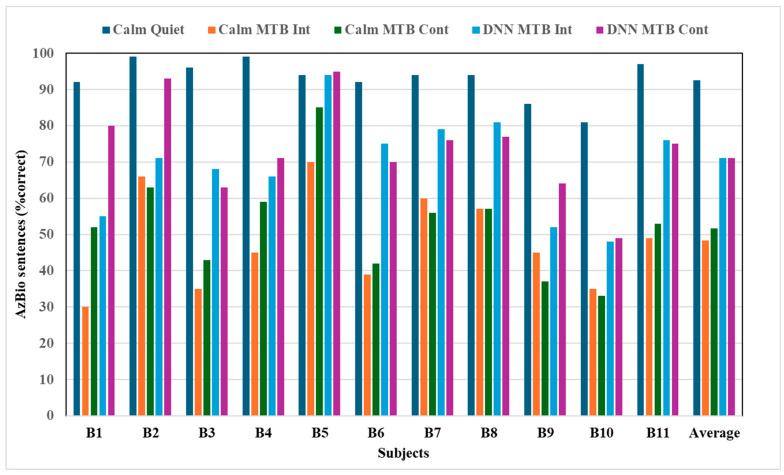
Individual and mean speech perception scores for Group 2 (N = 11). Performance is compared across the Calm Situation and DNN-based noise reduction programs in quiet, as well as in continuous and interrupted multi-talker babble.

Figure 4 presents speech perception scores for Group 2, comparing the continuous and interrupted MTB scores for the “Calm Situation” program to the DNN-based noise reduction program. Using the Calm program, participants averaged 93% in quiet, dropping to 48% (interrupted MTB) and 53% (continuous MTB). In contrast, the DNN-based noise reduction program improved speech perception, yielding higher average scores of 69% and 74% in interrupted and continuous noise, respectively. On average, mean scores were slightly higher in continuous MTB compared to interrupted MTB for both programs. The data met the assumptions for normality (Shapiro–Wilk test). A one-way ANOVA confirmed a statistically significant difference in sentence recognition across conditions [F (4, 50) = 22.80, *p* < 0.001]. Subsequent pairwise comparisons using the Student–Newman–Keuls post hoc test revealed significant differences (*p* < 0.001) in all test conditions, except no significant differences were found between interrupted and continuous noise for the Calm Situation (*p* = 0.406) or the DNN-based noise reduction (*p* = 0.416) programs. Despite the lack of significant group effects, individual data analysis revealed notable performance variations. For instance, Patient 1 demonstrated a substantial improvement in continuous MTB versus interrupted MTB across both programs. In contrast, Patients 2 and 9 showed this improvement only when using the DNN-based program. Conversely, Patients 4 and 5 performed better in continuous MTB solely within the manual “Calm Situation” program. These results are discussed in the following section.

## 4. Discussion

This study evaluated the impact of interrupted versus continuous MTB noise on speech perception across two groups of bimodal CI users, all of whom were fit with a Phonak Audéo Sphere Infinio hearing aid in their non-implanted ear. For Group 1, testing was conducted using the automated programs in both the CI and the hearing aid. Because the speech stimuli and MTB noise were co-located at 0 degrees azimuth, the beamforming features were not expected to influence outcomes. However, the noise reduction algorithms—particularly the DNN-based processing in the hearing aid—were able to activate due to the sufficient duration of noise that was provided. Once activated, DNN-based processing improves the SNR and thus significantly improves speech perception benefit [4,7,8,9].

The results from Group 1 demonstrated a clear performance gap between noise types: participants experienced a 50 percentage-point decrease in speech perception when tested in interrupted noise compared to only a 21 percentage-point decrease in continuous noise. This indicates that continuous noise yielded a 29 percentage-point higher speech perception score than interrupted MTB, highlighting the critical role of algorithm activation time in bimodal performance. These results strongly support the use of continuous noise over the standard interrupted noise protocols routinely used to assess implant candidacy and post-operative speech perception outcomes in CIs.

In addition to hearing aid noise processing, one must also consider the role of auditory stream segregation for speech perception in noise. Continuous noise provides the listener with sufficient time to acclimatize to the acoustic environment, potentially facilitating this aspect of the ‘cocktail party effect’ and improving focus on the primary speech stimulus.

To investigate the role of auditory stream segregation and acclimatization, a second group of bimodal CI users was evaluated (i.e., Group 2). These participants used their default CI programs alongside two manual hearing aid settings: the “Calm Situation” (no noise reduction) and a DNN-based noise reduction program (active noise reduction). The results revealed a clear contrast in performance across conditions. Compared to the quiet condition, the introduction of interrupted and continuous noise resulted in a 45 and 40 percentage-point decrease in speech perception, respectively, when the hearing aid was set to the Calm Situation. With the DNN-based program, performance remained significantly higher; the introduction of interrupted and continuous noise resulted in only a 24 and 19 percentage-point decrease compared to the baseline quiet scores. As expected, the mean differences between interrupted and continuous noise did not reach statistical significance for either program. This finding is unsurprising, as the hearing aid was set to a manual program and therefore the noise processing was stable across the two noise conditions.

Of interest is the high inter-subject variability observed in Group 2. Specifically, users 1, 2, 4, 5, and 9 exhibited substantial individual differences in performance between the two noise types with the Calm Situation and/or DNN-based noise reduction program. These results suggest that certain individuals are better equipped to utilize auditory stream segregation [10] when exposed to continuous background noise. The continuous background noise test condition, which more accurately reflects real-world environments, allows listeners to leverage auditory stream segregation to isolate and focus on the target signal.

Using interrupted noise in speech perception testing likely yields scores that fail to reflect a patient’s real-world performance. Because the noise is intermittent, it lacks the duration required for hearing aid algorithms to fully engage and optimize the Signal-to-Noise Ratio (SNR). Additionally, these interruptions hinder the listener’s ability to utilize auditory stream segregation.

By exaggerating listening difficulties, assessments using interrupted noise systematically underrepresent the true benefit of the device. This discrepancy leads to suboptimal programming decisions and may inadvertently discourage clinicians from prescribing advanced technology based on misleadingly low performance metrics.

Of note, the average ages of study participants in Group 1 and Group 2 were 81.4 and 78.3 years old, respectively. Auditory processing speed slows with increasing age [15,16]. Thus, the high inter-subject variability observed in Group 2 may be related to their advanced age. However, this data suggests that the use of continuous noise in clinical speech perception testing is especially imperative in the elderly population due to this decline in central processing.

Overall, the findings from this study support the shift toward using continuous noise in clinical assessments of speech perception. Not only does continuous noise allow noise reduction algorithms in hearing devices to activate and improve the SNR, but it also aids in auditory stream segregation for at least some hearing-impaired listeners. Because continuous noise represents natural listening conditions more faithfully than interrupted noise, it should be the preferred stimulus used for evaluating speech perception and device benefit in hearing-impaired populations.

## 5. Conclusions

This study evaluated speech perception in bimodal cochlear implant users across three conditions: quiet, interrupted, and continuous MTB noise. Both automatic and manual DNN-based noise reduction programs were assessed. Results indicated significantly higher speech perception scores in continuous MTB compared to interrupted MTB when using the automatic program. This suggests that continuous noise provides a sufficient temporal window for the algorithm to stabilize, effectively improving the SNR. In contrast, results for the manual programs were more variable across subjects. This variability suggests that some bimodal users may need more time to analyze a noisy environment before they can successfully employ auditory stream segregation. In continuous noise, these listeners are better able to focus on the target signal and suppress background interference, finally achieving the selective attention required for the ‘cocktail party’ effect.

## Data Availability

The original contributions presented in this study are included in the article. Further inquiries can be directed to the corresponding author.
